# The family Lohmanniidae (Acari, Oribatida) II: two new Oribatid mites, *Meristacarus perikopesis* sp. n. from Costa Rica and *Torpacarus eidikoterai* sp. n. from Kenya

**DOI:** 10.3897/zookeys.743.22815

**Published:** 2018-03-14

**Authors:** Nestor Fernandez, Pieter Theron, Sergio Leiva, Anine Jordaan

**Affiliations:** 1 National Council of Scientific and Technological Research, Argentina (CONICET), Subtropical Biological Institute (IBS), Evolutionary Genetic Laboratory FCEQyN, Misiones National University, Felix de Azara 1552, 6º, (3300) Posadas Misiones, Argentina; 2 Research Unit for Environmental Sciences and Management, North-West University, Potchefstroom Campus, 2520, South Africa; 3 Fellowship, National Institute Agricultural Technology (INTA), Experimental Rural Agency, Aimogasta, La Rioja, Argentina; 4 Laboratory for Electron Microscopy, North-West University, Potchefstroom Campus, 2520, South Africa

**Keywords:** Costa Rica, Kenya, *Lohmanniidae*, systematics

## Abstract

Two very particular new species of the family Lohmanniidae were studied and described using optical and Scanning Electron Microscopy (SEM). *Meristacarus perikopesis* **sp. n.** displays complex cuticular microsculpture with cross-shaped grooves and pusticulate porose areas; ten transversal bands, with reticulate-foveate microsculpture; *S*_4_, *S*_5_, *S*_7_, *S*_10_ not crossing medial notogastral plane, amongst other characters. *Torpacarus eidikoterai* **sp. n.** with: prodorsum - rostrum weakly bilobate with small central structure and CSO present. Six transversal depressions present, transversal bands absent; but six transversal depressions present, none of the depressions crossing medial notogastral plane.

## Introduction

In this paper we continue our study of material collected in the Turrialba forest of Costa Rica, and embark on the analysis of an extensive collection of material from Kenya. A brief discussion of previous studies of these genera is given below.

The genus *Meristacarus* was discovered and described by Grandjean in 1934 and today consists of more than fifteen species and three subspecies with worldwide distribution (Subias 2017). Authors contributing further knowledge include Aoki (1965); Balogh (1958, 1961); Balogh and Balogh (1983); Canestrini (1897); Clement (1995); Corpus-Raros (1979); Corpus-Raros and Lit (2009); Csiszar (1961); Grandjean (1934); Hammer (1979); Haq and Jaikumar (1993); Haq and Clement (1995); Mahunka (1978, 1988); Peres-Iñigo (1969).

The genus *Torpacarus* was described by Grandjean in 1950 and comprises 14 species worldwide (Subias 2017) studied by amongst others: Balogh and Mahunka (1981); Mac Daniel et al. (1979); Mahunka (1983); Schatz (1984, 1994); Stary (1998); Wallwork (1962).

Two studies by Alberti et al. (1996, 1997), conducted using SEM and TEM (Transmission Electron Microscopy), included the Lohmanniidae, and referred specifically to *Mixacarus* and *Meristacarus*. These studies greatly assisted in the study reported on in this paper.

## Materials and methods

Specimens studied by means of optical microscopy followed the techniques described by Grandjean (1949) and Krantz and Walter (2009). Specimens studied under SEM followed the techniques of Alberti and Fernandez 1988, 1990a, 1990b; Alberti et al. 1991, 1997; Fernandez et al. 1991. Equipment used was identical to previous studies (see Fernandez et al. 2017).

Optical drawings should be considered semi–schematics with regard to cuticular microsculpture and setal shapes. SEM micrographs provide much higher levels of precision and detailed figures.

Body measurements taken: total length (tip of rostrum to posterior edge of notogaster); width (widest part of notogaster). All measurements given in micrometers (μm). Measurements of setae taken on three specimens (SEM); length of setae are to be considered provisional as, though preservation was good, these mites were preserved in alcohol for over 35 years and possible damage to setal tips cannot be excluded.

Optical microscopy (standard, polarized and phase contrast) was used during leg chaetotaxy studies. Setal formulae of the legs include the number of solenidia (in parentheses); tarsal setal formulae include the famulus (ε).

### Morphological terminology and abbreviations

Morphological terms and abbreviations used are those developed by Grandjean (1928–1974) (cf. Travé andVachon 1975; Norton and Behan-Pelletier (in Krantz and Walter 2009); Fernandez et al. 2013a, b, c). For setal types Evans 1992:73 and for ornamentation of cuticular surfaces Murley 1951(in Evans *op.cit*: 9) were used.

Additional abbreviations for *Torpacarus seidikoterai*: **at** anterior transversal depression; **mt_1_** medial transversal depression, situated posterior to **mt**; **pt_2_** posterior depression situated behind **pt** depression.

Institution abbreviation. **MNHG**: Museum of Natural History, Geneva, Switzerland.

## Taxonomic part

### Family Lohmanniidae Berlese, 1916

#### Genus *Meristacarus* Grandjean, 1934

##### Meristacarus perikopesis sp. n.

Taxon classificationAnimaliaORDOFAMILIA

http://zoobank.org/4838F7D1-79AA-44E9-BC1E-EB3DFBF13145

Figures 1–7 , 8–10 , 11–13 , 14–18 , 19–22 , 23–28

###### Etymology.

The specific epithet *perikopesis* is derived from perikopés, (Περικοπές in Greek meaning cuts in English) by the characteristic cross-cut grooves present on the cuticle.

###### Type material.

***Holotype***.♀“CR 0978 Tu 15 Costa Rica Turrialba foret naturelle du Catie alt.560 m. Triage d’humus pied arbre à contreforts. 11. IX. 1978. LEG P.WERNER”; material conserved in 70 % ethanol, deposited in MHNG. ***Paratypes***. 2 ♀♀, same data and locality, deposited in MHNG; preserved in 70 % ethanol.

###### Diagnosis

(adult female). Integument. Very complex with irregular cross-shaped grooves on prodorsum and notogaster. Smooth surfaces: anterior zone prodorsum extending to rostrum; zone posterior to *b.sj*; subcapitulum; anterolateral zone of prodorsum; epimeral zones; genital plate; preanal plate. Pusticulate surfaces (porose areas) on prodorsum, notogaster. Reticulate–foveate: Sb, S_1_, S_2_, S_3_, S_4_, S_5_, S_6_, S_7_, S_8_, S_9_, S_10_ transversal bands, notogaster. Colliculate: basal subcapitulum region; areas surrounding 1a epimeral setae; v.sj; anal-adanal zone and BPDA; legs I, II, III, IV. Undulate on lateral epimeral zone. Punctate: entire prodorsum and notogaster. Sulcate: bothridial ring.

Prodorsum. Flat smooth chitinous edge, external to exa, exp, and le setae, postbothridial transverse band sb hardly discernible.

Notogaster. Sixteen pairs of primary notogastral setae: c_1_, c_2_, c_3_, d_1_, d_2_, d_3_, e_1_, e_2_, f_1_, f_2_, h_1_, h_2_, h_3_, p_1_, p_2_, p_3_;tentransversal bands: S_1_, S_2_, S_3_, S_4_, S_5_, S_6_, S_7_, S_8_, S_9_, S_10_. Transversal bands *S*_4_, *S_5_, S_7_, S_10_* not crossing medial notogastral plane; prodorsal, notogastral setae barbate; adoral setae: or_1_ large, teardrop-shaped; or_2_ elongate, wide acutely terminating tip; or_3_ long, thin, sharply tipped.

###### Description

**(Adult female).** *Measurements* 887 (876–920) × 376 (351–386) μm (n: 3).

**Figures 1–7.**
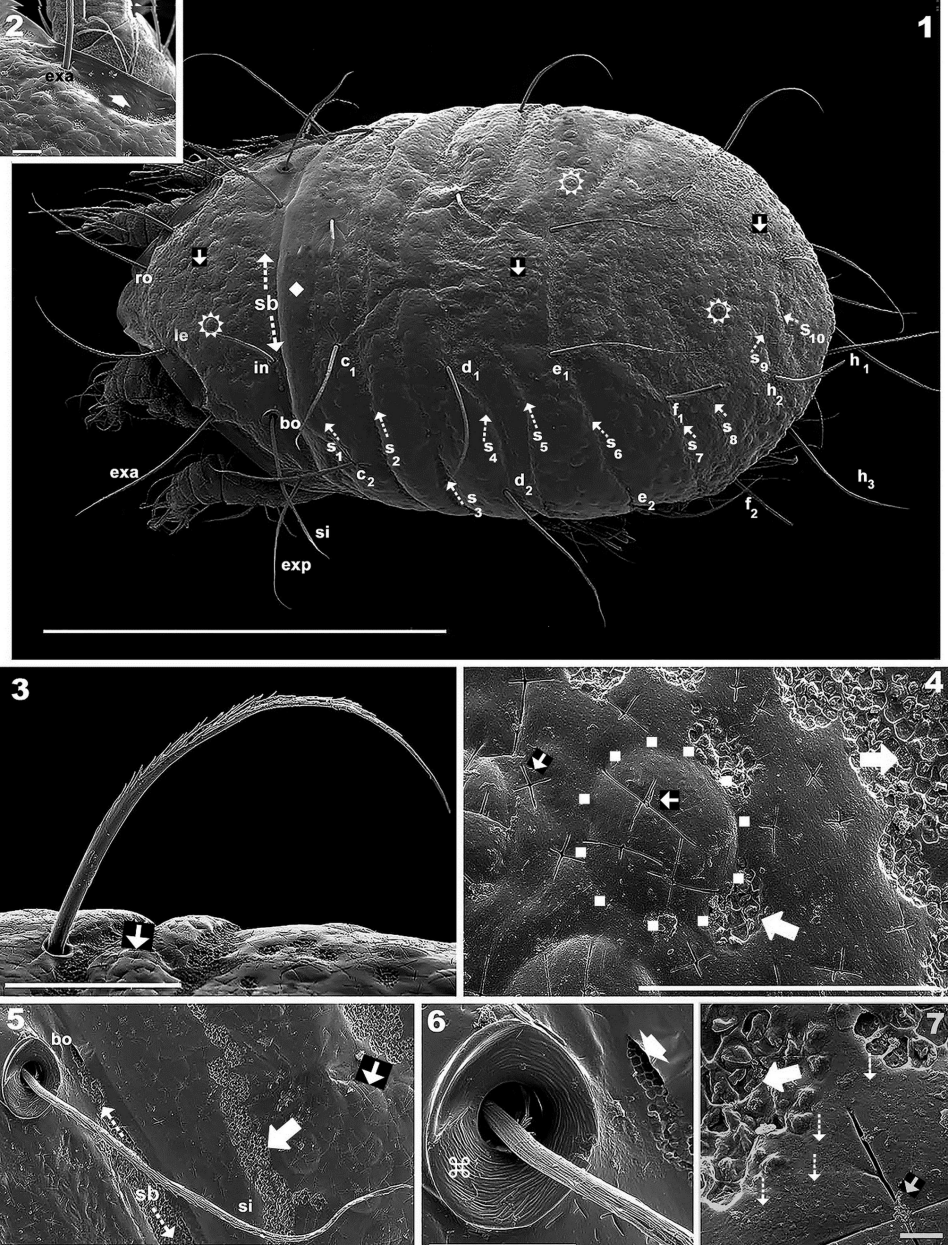
*Meristacarus perikopesis* sp. n. Adult with cerotegumental layer. SEM micrographs. **1** dorsal view **2** dorsal prodorsal margin **3** notogastral setae, lateral view **4** cuticular microsculpture **5** sensillus, general view **6** bothridium detail **7** cuticular microsculpture, high magnification. Abbreviations: See “Material and methods”. Scale bars: 500 μm (**1**); 20 μm (**2**); 50 μm (**3**); 30 μm (**4**); 100 μm (**5**); **6** 20 μm (**6**); 2 μm (**7**).

*Shape.* Oval (Figure 1).

*Colour.* Yellow to light reddish yellow; slightly shiny when observed in reflected light.

*Cerotegument.* Mostly absent, on some regions (e.g., near the bothridium (Figure 6, indicated by solid upwards arrow) observed as very thick smooth layer; on some epimeral zones and legs, gives the impression of randomly distributed dust (Figures 14, 26, indicated by white and black upwards white bar arrow). The cerotegumental layer was most probably degraded during the long period of preservation in alcohol.

**Figures 8–10.**
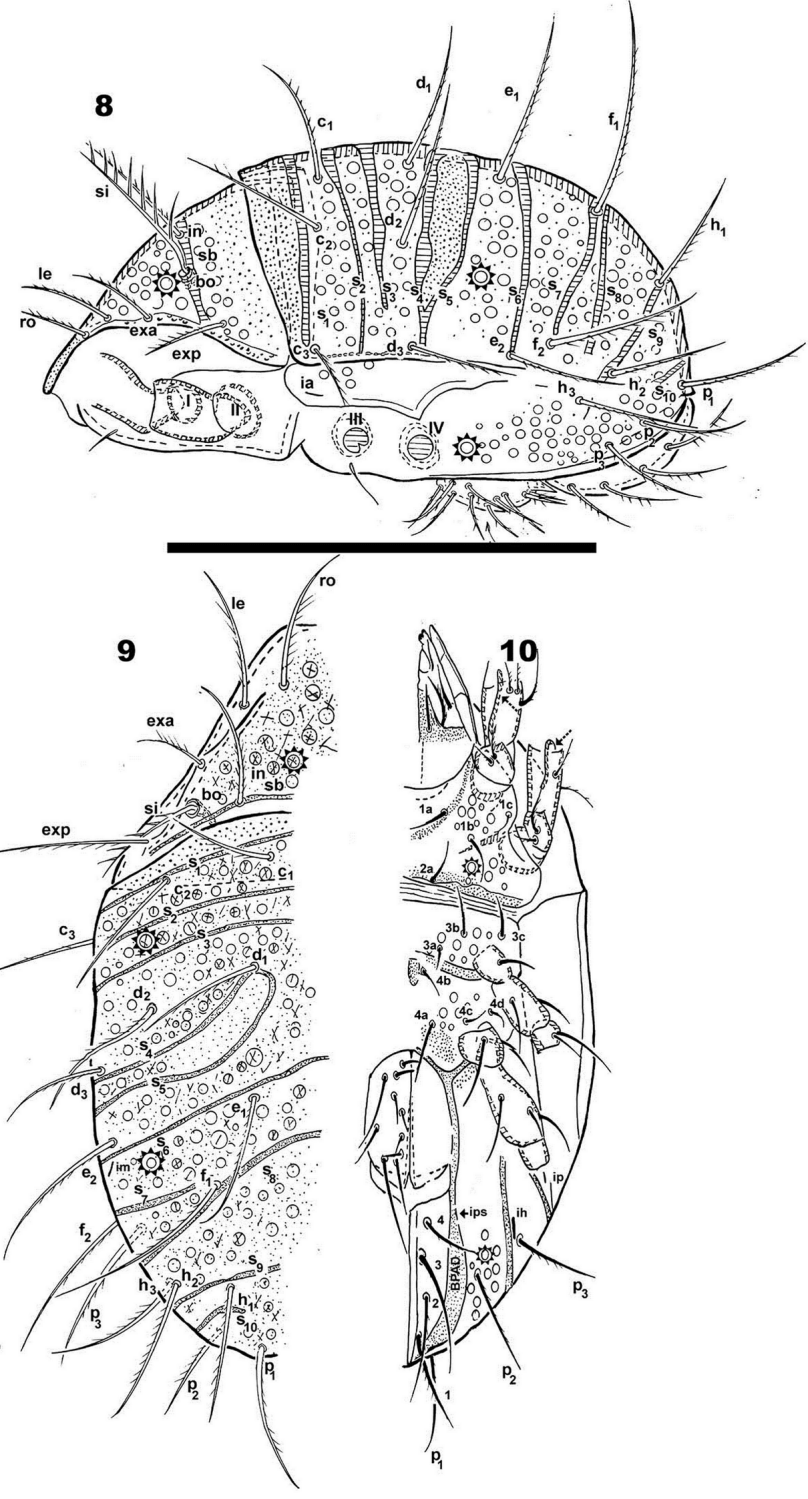
*Meristacarus perikopesis* sp. n. Adult, optical microscopy. **8** lateral view **9** dorsal view **10** ventral view. Scale bar: 600 μm.

*Integument.* Very complex. *Irregular cross-shaped grooves* (Figures 1, 3, 4, 5, 6, 13, indicated by solid leftwards arrow) present on entire prodorsum and notogaster, as well as on pusticulate surfaces. *Smooth* surfaces, anterior zone of prodorsum up to rostrum (Figure 12 indicated by solid rhombus); zone posterior to b.sj (Figure 1 indicated by solid rhombus); subcapitulum (Figures 14, 19, 21 indicated by solid rhombus); anterolateral region of prodorsum (Figure 2 indicated by solid rhombus); epimeral zone (Figure 14 indicated by solid rhombus); genital plate (Figure 15 indicated by solid rhombus); preanal plate (Figure 16 indicated by solid rhombus). *Pusticulate surfaces*: prodorsum, notogaster, and epimeral region (Figures 1, 8, 9, 10, 12, 13 indicated by white sun with rays) (See Remarks); pustules between 7–20 μm in diameter (Figure 4, pustules indicated by surrounding small squares). *Reticulate–foveate* on Sb, S_1_, S_2_, S_3_, S_4_, S_5_, S_6_, S_7_, S_8_, S_9_, S_10_ (Figures 5, 23 indicated by outlined rightwards arrow). This microsculpture is also observed, irregularly distributed, in vicinity of pustules (Figure 4 indicated by outlined rightwards arrow) and cross-shaped grooves (Figure 7 indicated by outlined rightwards arrow). *Colliculate*: subcapitulum basal zone; surrounding 1a epimeral setae (Figure 18 indicated by diamond shape made up of 4 smaller black diamonds); surrounding vsj (Figure 14 indicated by diamond shape made up of 4 smaller black diamonds); adanal plate and BPDA (Figure 16 indicated by) diamond shape made up of 4 smaller black diamonds; legs I, II, III, IV (Figures 23, 25, 26, 28, indicated by diamond shape made up of 4 smaller black diamonds). *Undulate*: lateral epimeral zone (Figure 14 indicated by interrupted outlined arrow); *punctate* on prodorsum and notogaster (Figure 7 indicated by narrow dashed arrow); *sulcate*: bothridial ring (Figure 6).

*Setation* (legs not included). *Simple, smooth*: epimeral (Figures 14, 18); subcapitular a (Figure 20); genital (Figure 15). *Simple, barbed*: prodorsum (Figures 12, 13), notogaster (Figure 3); subcapitular h, m_1_, m_2_ (Figure 19); adanal (Figure 17). Numerous long barbs, principally situated on one side of the seta (Figures 3, 13), sometimes on the opposite side, a few very small barbs can be observed.

**Figures 11–13.**
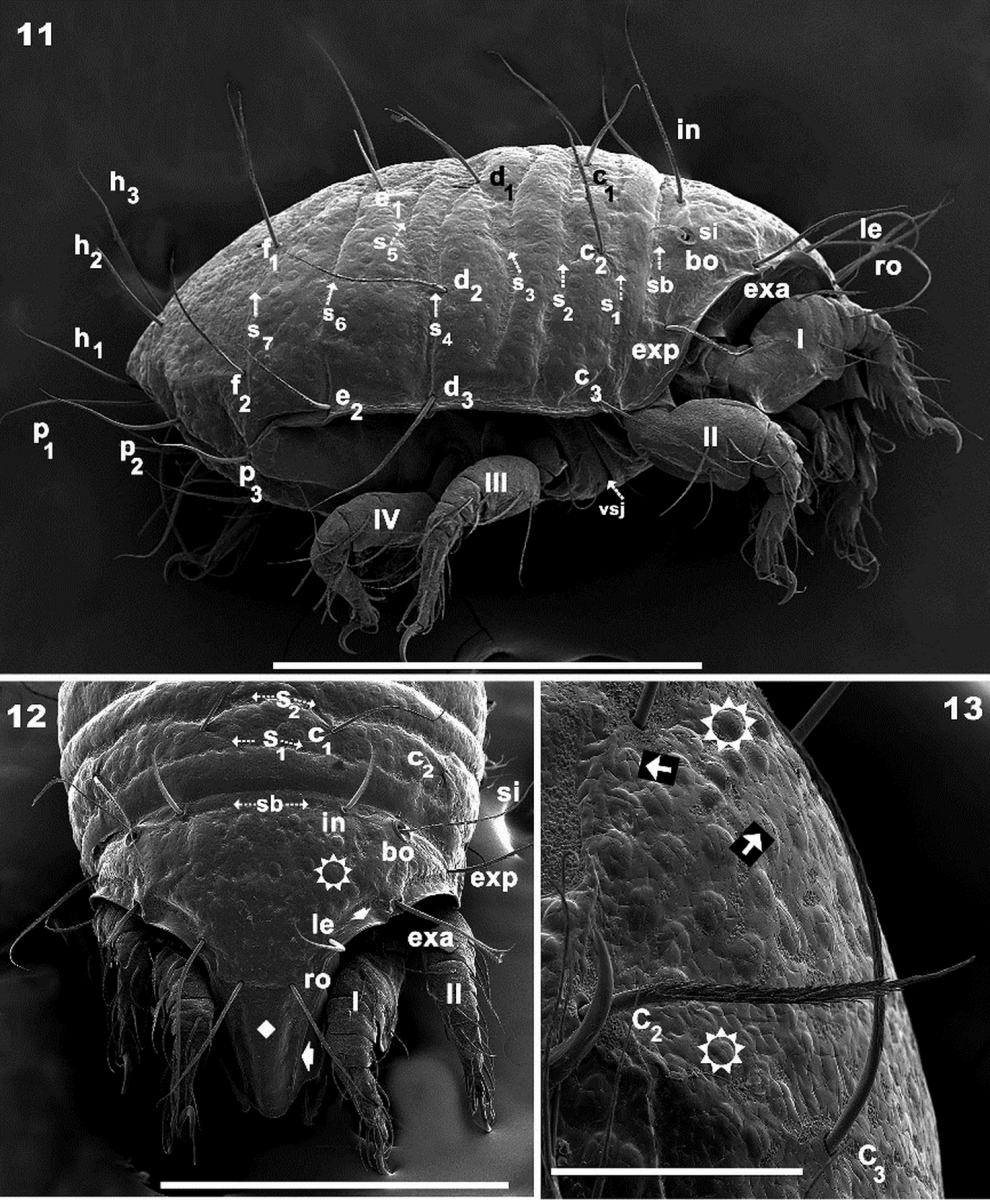
*Meristacarus perikopesis* sp. n. Adult with cerotegumental layer. SEM micrographs.**11** lateral view **12** frontal view **13** notogastral anterior lateral view. Scale bars: 500 μm (**11**); 400 μm (**12**); 100 μm (**13**).

**Figures 14–18.**
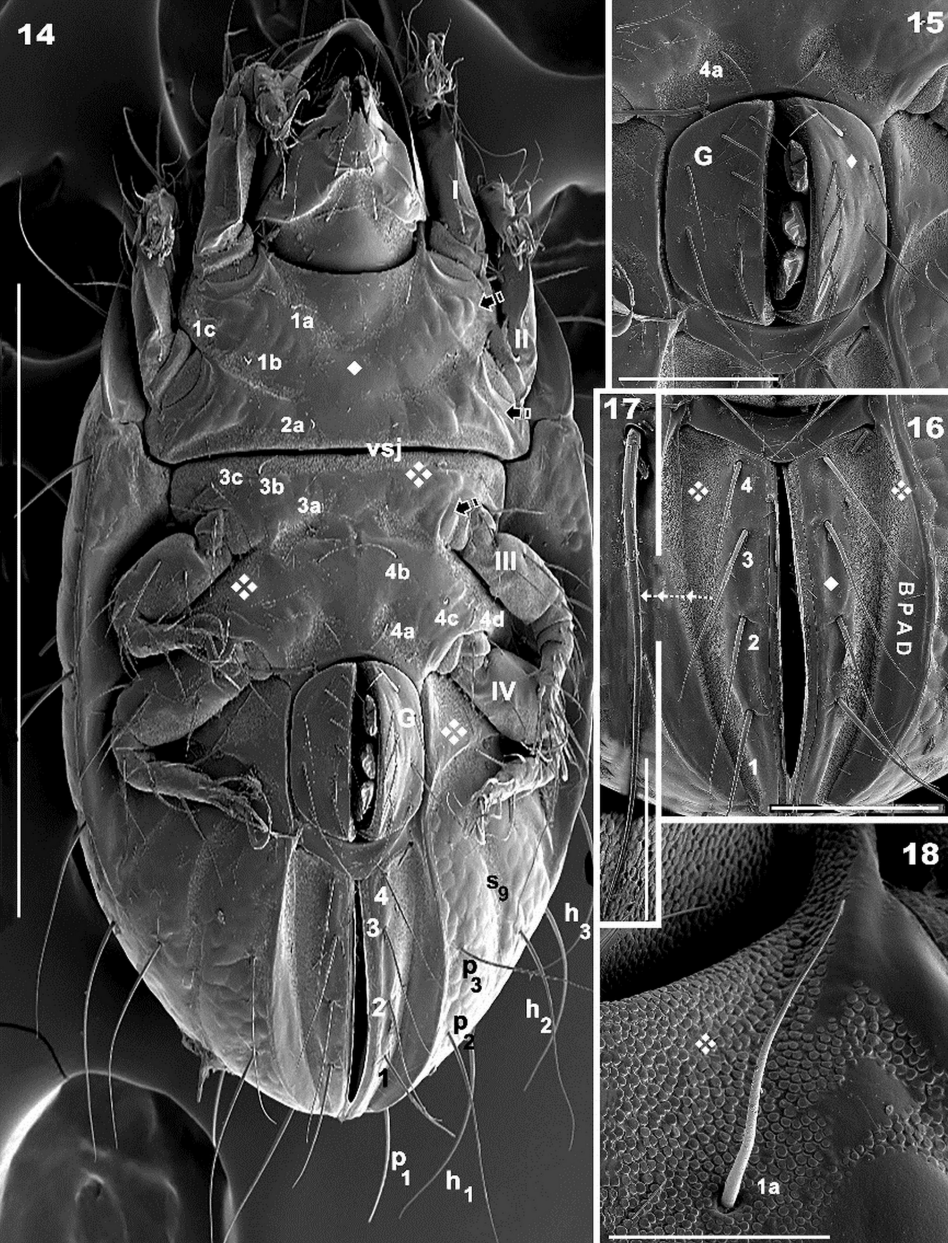
*Meristacarus perikopesis* sp. n. Adult with cerotegumental layer. SEM micrographs. **14** ventral view **15** genital zone **16** ano-adanal region **17** adanal setae **18** epimeral setae 1a. Scale bars: 500 μm (**14**); 100 μm (**15**); 100 μm (**16**); 25 μm (**17**); 30 μm (**18**).

*Prodorsum*. Shape: triangular, rounded apex in dorsal view (Figures 1, 9); triangular in lateral view (Figures 8, 11); in frontal view, triangular with curved sides (Figure 12). Rostrum ovoid (Figures 12, 14); flat smooth chitinous edge present on either side of prodorsal area, externally to exa, exp, and le setae, derived from margin of lateral depression housing legs (Figures 2, 12, indicated by solid upwards arrow); region between level of rosetal insertion and rostrum, smooth, elongate, clearly delimited by flat chitinous edge (Figure 12 indicated by solid rhombus); ro setae large, length 155 (148–171) μm directing forward (Figures 1, 11, 12); le setae directing forward, length 200(196–221) μm (Figure 1, 12); in setae upright, 91 (86–101) μm; exa setae 201(195–228) μm ; exp setae 91 (87–98) μm .

*Bothridium (bo)* ring-shaped, rounded, with particular microsculpture slightly elevated from the cuticular surface (Figures 5, 6), opening directed upwards with slight lateral tilt. Sensillus (*Si*) pectinate, some pectines elevated but most slanted along main body of *Si* (Figures 1, 5). Si stem longitudinally sulcate (Figure 6). Postbothridial transverse band *sb* hardly discernible, situated posterior to bo; in setae situated on *sb* margin (Figures 1, 5, 11, 12).

*Notogaster.* Sixteen pairs of primary notogastral setae clearly discernible: c_1_, c_2_, c_3_, d_1_, d_2_, d_3_, e_1_, e_2_, f_1_, f_2_, h_1_, h_2_, h_3_, p_1_, p_2_, p_3_ (Figures 1, 8, 9, 11). Ten transversal bands: S_1_, S_2_, S_3_, S_4_, S_5,_ S_6_, S_7_, S_8_, S_9_, S_10_ (Figures 1, 5, 8, 9, 11). S_1_ anterior to setae c_1_ ,c_2_, c_3_, crossing transverse medial notogastral plane (Figures 1, 8, 9, 11, 12); S_2_ crossing transverse medial notogastral plane, extending slightly beyond c_2_ setae, terminating near c_3_ in a large ovoid tip; S_3_ situated behind c setal alignment and in front of d setal alignment, crossing medial notogastral plane (Figure 11); S_4_ behind d_1_, d_2_ setal alignment, not crossing medial notogastral plane;S_5_ oblique, exceeding d_1_ setal insertion level, terminating in rounded end (Figures 1, 8, 9, 11), not crossing medial notogastral plane; S_6_ crossing medial notogastral plane, thin band surpassing e_2_ setal insertion level, laterally extending to unsclerotized lateral longitudinal line; S_7_ at level f_1_, f_2_ setal insertion, not crossing medial notogastral plane, terminating near f_1_ setal insertion; S_8_ behind f_2_ setal level, extending obliquely to f_1_ setal insertion, crossing medial notogastral plane; S_9_ situated at h setal insertion level, crossing medial notogastral plane; S_10_ in front of p_1_ insertion level, not crossing medial notogastral plane.

Five pairs of lyrifissures present: ia, ip, im, ips, ih; ips situated on adanal fold band (BPDA) (Figure 10); im near e_2_ setae and ih behind p_3_.

*Lateral region*. In lateral view certain transverse bands are hardly discernible, principally S_8_, S_9_, S_10_ (Figure 11). Flat smooth prodorsal margin present on either side of cavities housing legs I-IV when retracted. Anterior notogastral zone presenting conspicuous tectum and clearly defined unsclerotized lateral longitudinal line (Figure 8).In posterior notogastral zone, where unsclerotized line is absent, notaspis and pleuraspis not delimited (Figure 8).

**Figures 19–22.**
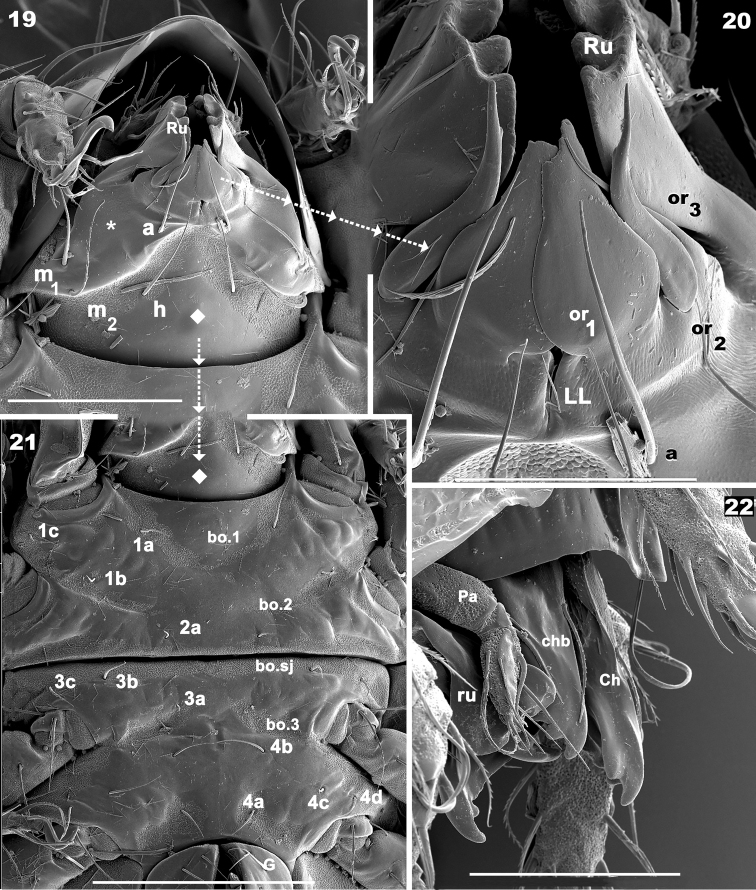
*Meristacarus perikopesis* sp. n. Adult female with cerotegumental layer. SEM micrographs. **19** subcapitulum **20** adoral setae **21** epimeral zone **22** rostrum and anterior zone of chelicera and palp. Scale bars: 100 μm (**19**); 40 μm (**20**); 200 μm (**21**); 100 μm (**22**).

*Ventral region*. Subcapitulum more or less triangular, posterior zone ovoid. Four pairs of subcapitular setae, a, m_1_, m_2,_ h. Smooth elevated triangular structure (Figure 19 indicated by*) containing m_2_, m_1_, a setae determined by oblique line from subcapitular marginal zone to medial longitudinal plane, with h setae situated outside triangle in a depressed area (Figure 19). Cuticle smooth in central zone of this depressed area, but colliculate microsculpture observed bordering the triangular elevated zone (containing m_2_, m_1_, h setae). Adoral setae (Figures 19, 20):or_1_ large, teardrop-shaped; or_2_ elongate, wide, terminating in acute tip; or_3_, long and thin, sharply tipped. Coxisternal region divided into two parts by ventrosejugal groove (Figures 14, 21). Apodemes clearly visible; epimeral setal formulae 3-1-3-4 (Figure 10). Genital plate rounded, undivided, usually with ten pairs of setae, but sometimes only nine pairs (Figures 14, 15); six simple setae paraxially and three or four antiaxially. Preanal plate more or less triangular, rounded central zone (Figures 14, 15, 16).

Adanal plate with four pairs of setae (Figure 16); setae with very small barbs (Figure 17). Band BPAD clearly visible; lyrifissure ips present near margin of this band (Figure 10).

**Figures 23–28.**
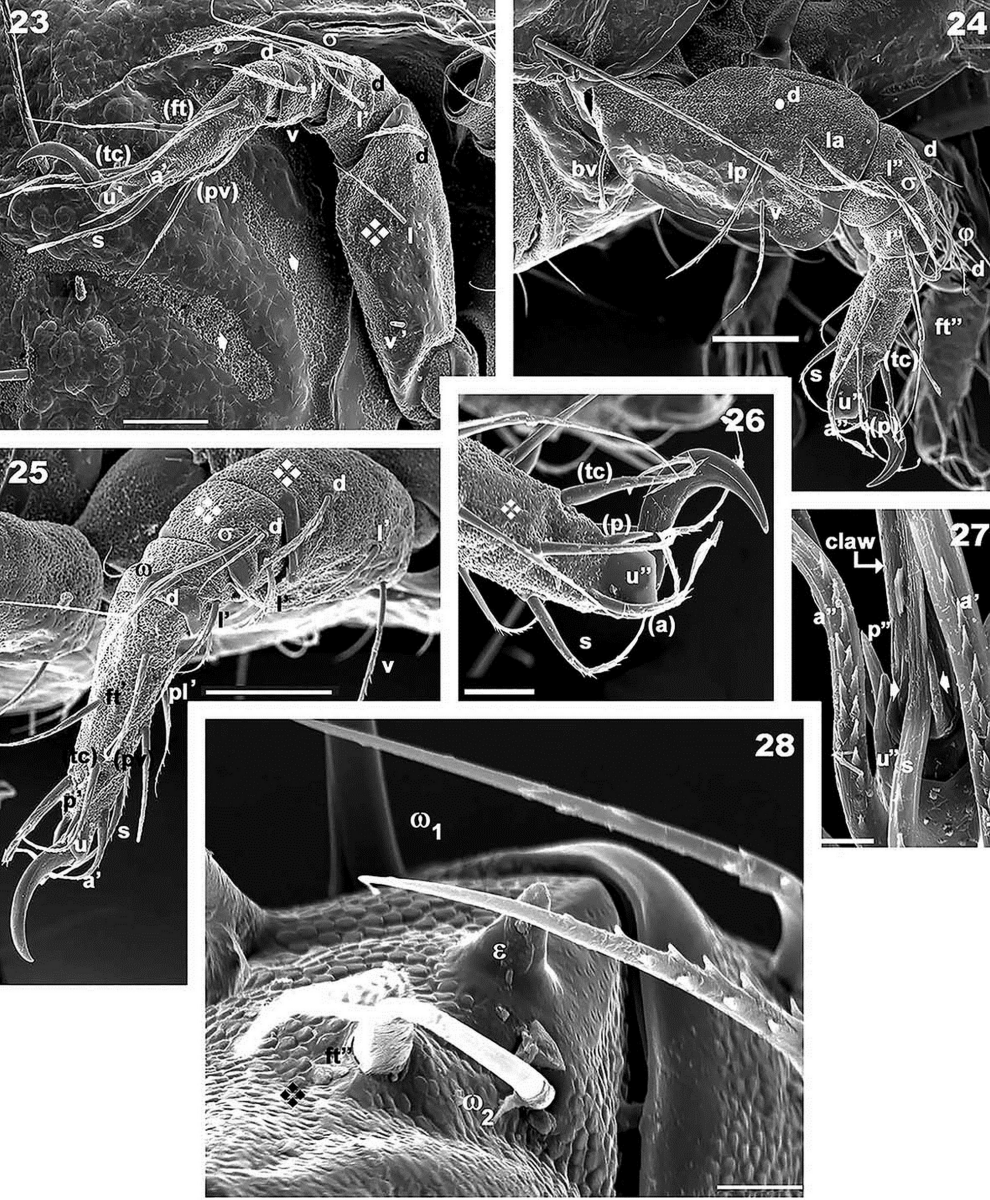
*Meristacarus perikopesis* sp. n. Adult female with cerotegumental layer. SEM micrographs. **23** leg IV paraxial view **24** leg II paraxial view **25** leg III **26** apical zone, leg II **27** ventral apical zone, leg II **28** tarsus I, famulus and solenidion zone. Scale bars: 50 μm (**23–25**); 20 μm (**26**); 5 μm (**27, 28**).

*Legs.* Two types of femora can be distinguished. Femora legs I and II displaying large ventral blade (Figure 14), femora legs III and IV with poorly developed ventral blade. Setae *u* laterally flattened on all legs (Figures 23, 24, 25, 26, 27), with flap housing *s* setae on claw (Figure 27 indicated by Solid upwards arrow). Famulus spur-shaped (Figure 28), setal formulae I (0-4-2-2-16-1) (2-1-2); II (0-5-4-4-15-1) (1-1-1); III (2-4-3-4-13-1) (1-1-0); IV (2-3-2-3-13-1(1-0-0).

###### Remarks.

**Figures 29–34.**
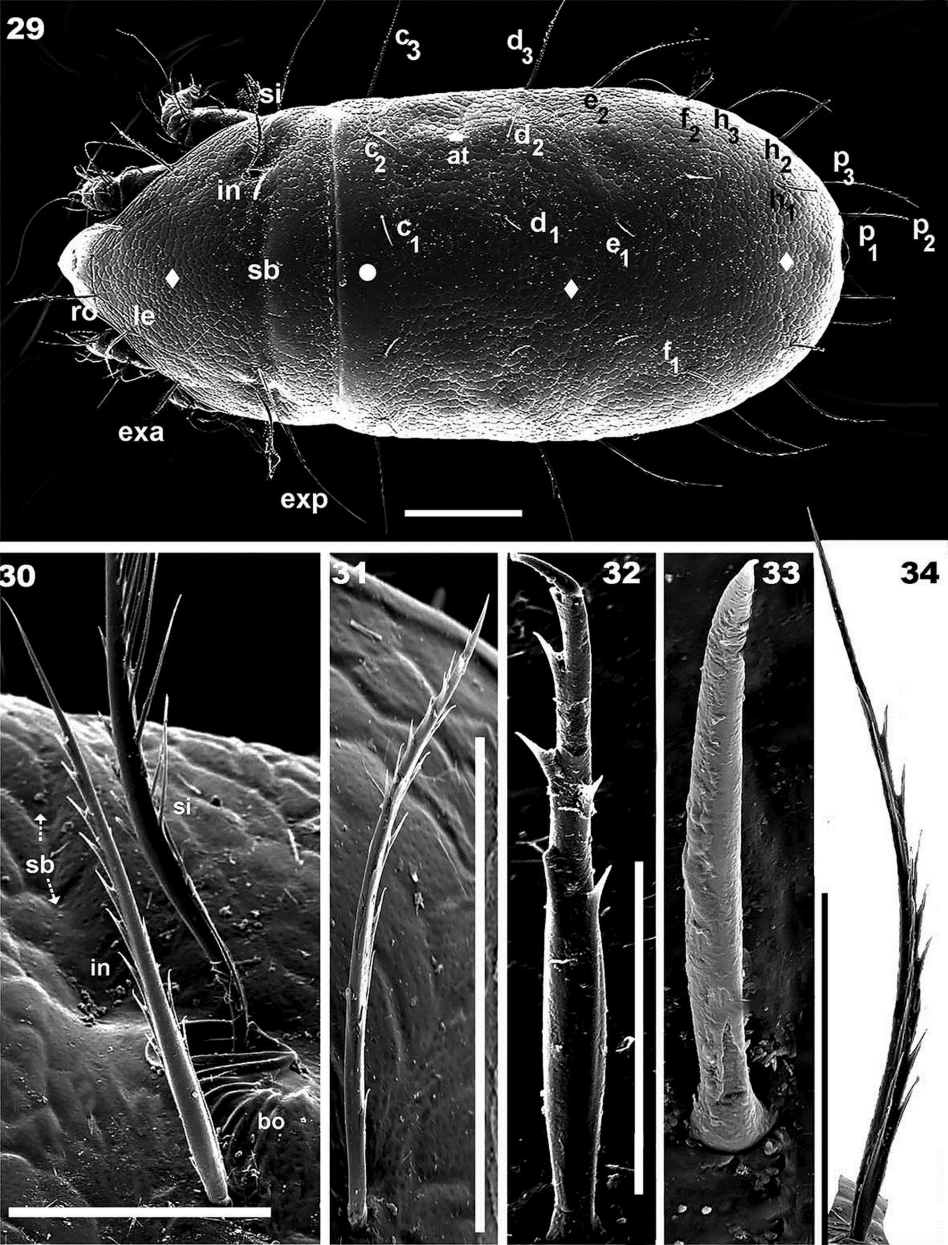
*Torpacarus eidikoterai* sp. n. Adult female with cerotegumental layer. SEM micrographs. **29** dorsal view **30** lateral view, interlamellar setae and sensillus **31** rostral setae **32** c_1_ notogastral setae **33** c_1_ notogastral setae, variations **34** notogastral setae, lateral view. Scale bars: 100 μm (**29**); 30 μm (**30**); 40 μm (**31**); 10 μm (**32**); 10 μm (**33**); 10 μm (**34**).

The porose area indicated by Grandjean 1934: 37 “…*et par sesairesporeuses au nombre de plusieurscentaines, répartiessurtoute la surface du corps*” describing *Meristacarus porcula*, is referred to as pusticulate cuticular microsculpture (diameter of pustules between 20–7μm) in this paper. These structures were studied using optical microscopy in order to confirm their similarity to the description given by Alberti et al. 1996: 280 “*In lohmanniid mites numerous distinct porose areas may be seen in certain taxa (e.g. Meristacarus, Mixacarus)*”. In 1997 Alberti et al. 1997: 58–63: indicated: “*In light microscopy they appear as distinct patches of higher transparency and show the fine striation perpendicular to the surface, that is typical of porose areas (Fig. 29)*” and referred to SEM studies: “*This similarity is also evident with SEM (Fig. 30 A) the porose areas are not visible from the exterior but very shallow depressions indicate sometimes their location*”.

Our results are markedly different to Alberti et al. 1997 (Fig. 30A) as, although the porose areas and/or shallow depressions are not visible under SEM, well-defined pustules are clearly discernible. Also, the *irregular cross-shaped grooves* differ from those described by Alberti et al. 1996, 1997, where reference was made to pores, for example Alberti et al. 1996: 281, indicates, with reference to function “*Such a glandular function is certain in the typical porose areas (octotaxic system, areas of part of the prosoma) in the medio dorsal porose areas of Acrogalumna males and the numerous areas of Mixacarus*” and “*The latter two types are remarkable in being innervated. The secretions pass through the inner layers of the epicuticle (no open pores) and are presumably lipids*” and later: “*This statement can now be modified in such a way that organs with one or another functions –respiration on the one hand or secretory on the other hand*…”.

#### Genus *Torpacarus* Grandjean, 1950

##### Torpacarus eidikoterai sp. n.

Taxon classificationAnimaliaORDOFAMILIA

http://zoobank.org/68C7E647-D2A8-4AEC-AACA-F0E1E7CE3501

Figures 29–34 , 35–37 , 38–41 , 42–46 , 47–52 , 53–54 , 55–58

###### Etymology.

The specific epithet *eidikoterai* is derived from eidikótera (ειδικότερα in Greek meaning particular in English), due to specimen characteristics.

###### Type material.

***Holotype***. ♀ Female KEN 77-42. Tana. River distr. Lac Shakababo près de Ngao. Tamisage broussailles avec des cactées. 28. X. 1977. LEG. V. Mahnert & J.L. Perret“. Material deposited in the Collection of the Museum of Natural History, Geneva. ***Paratypes***. same data, 2 ♀♀ deposited in MHNG; preserved in 70 % ethanol.

###### Diagnosis.

Microsculpture. Areolate: prodorsum, except for CSO zone; entire notogaster lateral to BPAD; near setal insertion p_2_, p_3_ extending to acetabulum IV; epimeral zone. Smooth: anterior prodorsal zone of CSO; anterior notogastral zone; anterior epimeral zone; central epimeral zone behind v.sj furrow; internal preanal zone; adanal plate. Colliculate: epimeral zone at level of acetabulum IV; around 4a epimeral setal insertion; lateral adanal zone and BPAD; elevated ridges on genital plate. Prodorsum. Rostrum weakly bilobate with small central structure; elevated smooth longitudinal zone with CSO; externally to exa, exp, le and ro setae, flat elevated margin extending dorsally, terminating near rostrum; ovoid ring-shaped bothridium, lateral opening; internal bothridial ring pronounced on cuticular surface; sensillus bipectinate; postbothridial transverse band sb forming shallow groove, transversal prodorsal band sb present. Notogaster. Sixteen pairs of primary notogastral setae: c_1_, c_2_, c_3_, d_1_, d_2_, d_3_, e_1_, e_2_, f_1_, f_2_, h_1_, h_2_, h_3_, p_1_, p_2_, p_3_; setae c_1_,c_2_ d_1_, d_2_ ,e_1_ either with few barbs, or nail-shaped. Transversal bands not observed, six paired depressions at, mt; nt, pt, mt_1_, pt_2_ present, depressions not crossing medial notogastral plane. Ventral region. Epimeral setal formulae (3-1-4-4).

**Description.** *Measurements.* SEM 756 (727–780) × 337 (281–400) μm (n: 8). Light microscopy: 775 (751–811) × 342 (334– 403) μm (n:4).

*Shape.* Elongate-oval (Figures 29, 36).

*Colour.* Specimens without cerotegument: brown-light red; slightly shiny when observed in reflected light.

*Cerotegument.* Not detected.

**Figures 35–37.**
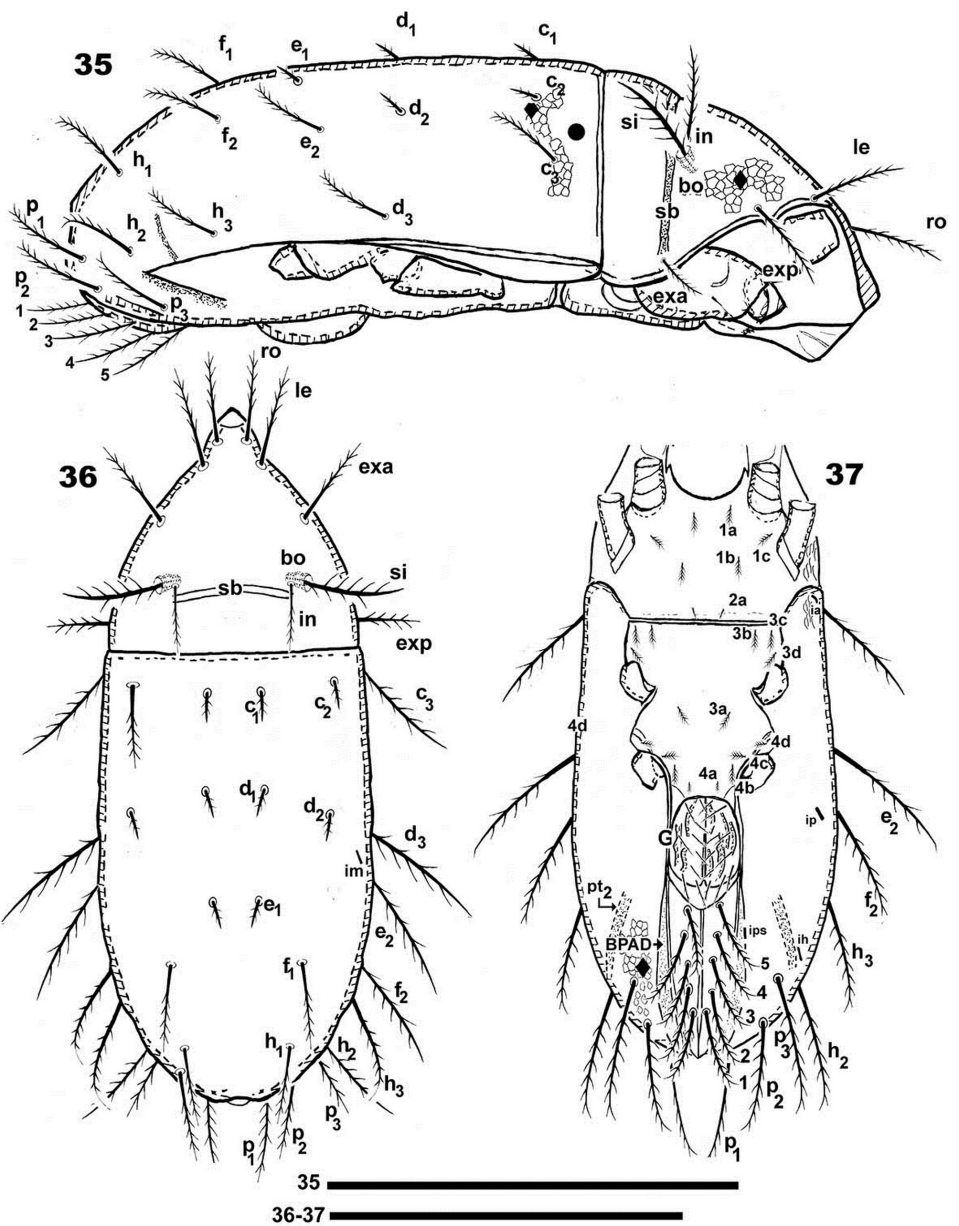
*Torpacarus eidikoterai* sp. n. Adult female, optical observations. **35** lateral view **36** dorsal view **37** ventral view. Scale bars: 399 μm (**35**); 500 μm (**36–37**).

**Figures 38–41.**
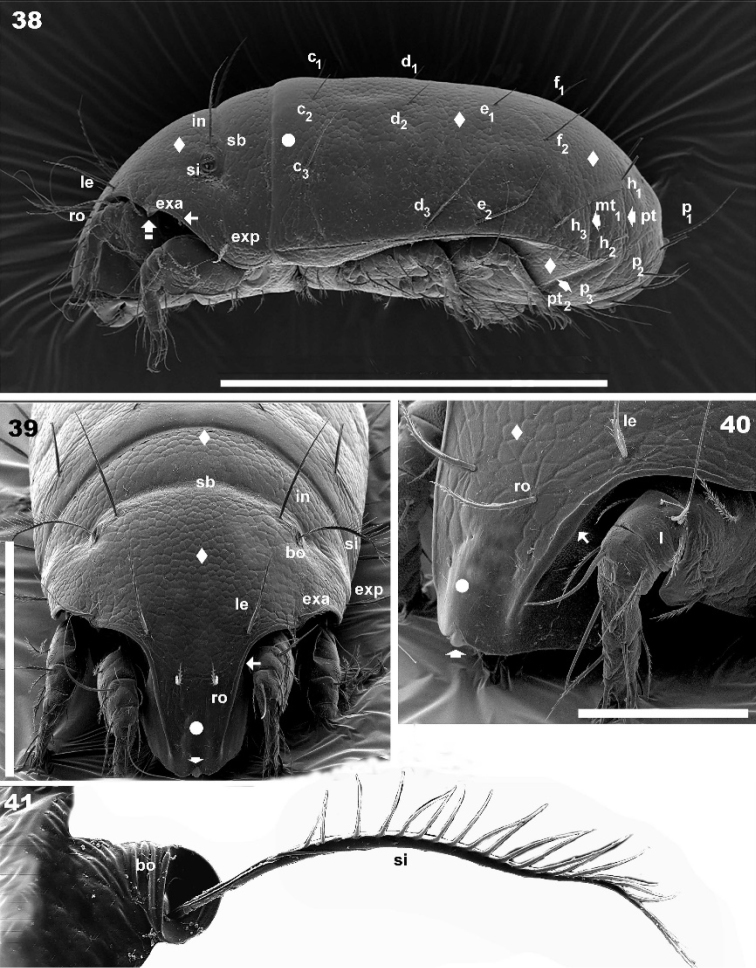
*Torpacarus eidikoterai* sp. n. Adult female with cerotegumental layer. SEM micrographs. **38** lateral view **39** frontal view **40** prodorsal anterolateral view **41** bothidia and sensillus, lateral view. Scale bars: 500 μm (**38**); 200 μm (**39**); 100 μm (**40**); 50 μm (**41**).

**Figures 42–46.**
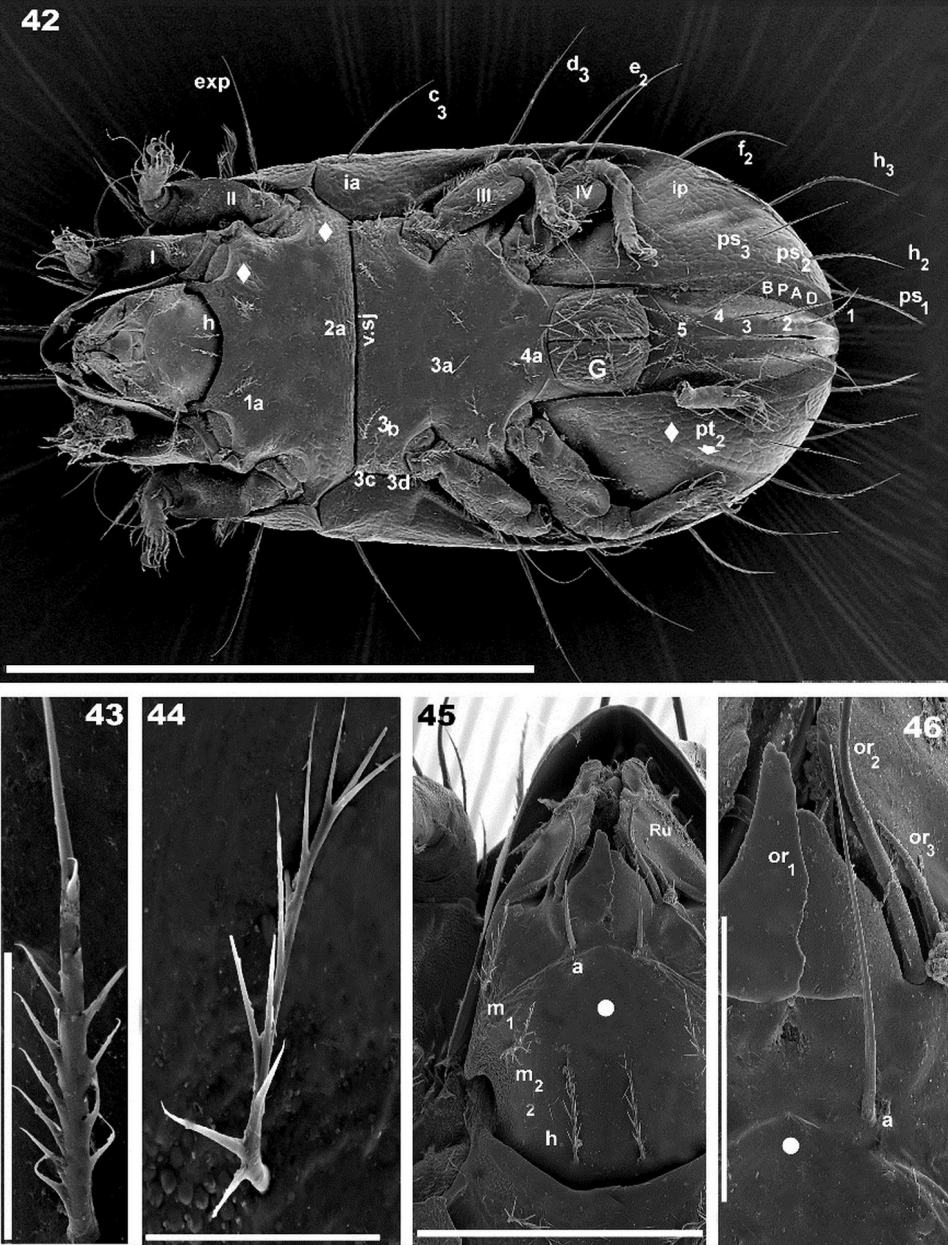
*Torpacarus eidikoterai* sp. n. Adult female with cerotegumental layer. SEM micrographs. **42** ventral view **43** subcapitular h setae **44** epimeral setae **45** subcapitulum ventral view **46** adoral setae. Scale bars: 500 μm (**42**); 20 μm (**43**); 20 μm (**44**); 100 μm (**45**); 30 μm (**46**).

**Figures 47–52.**
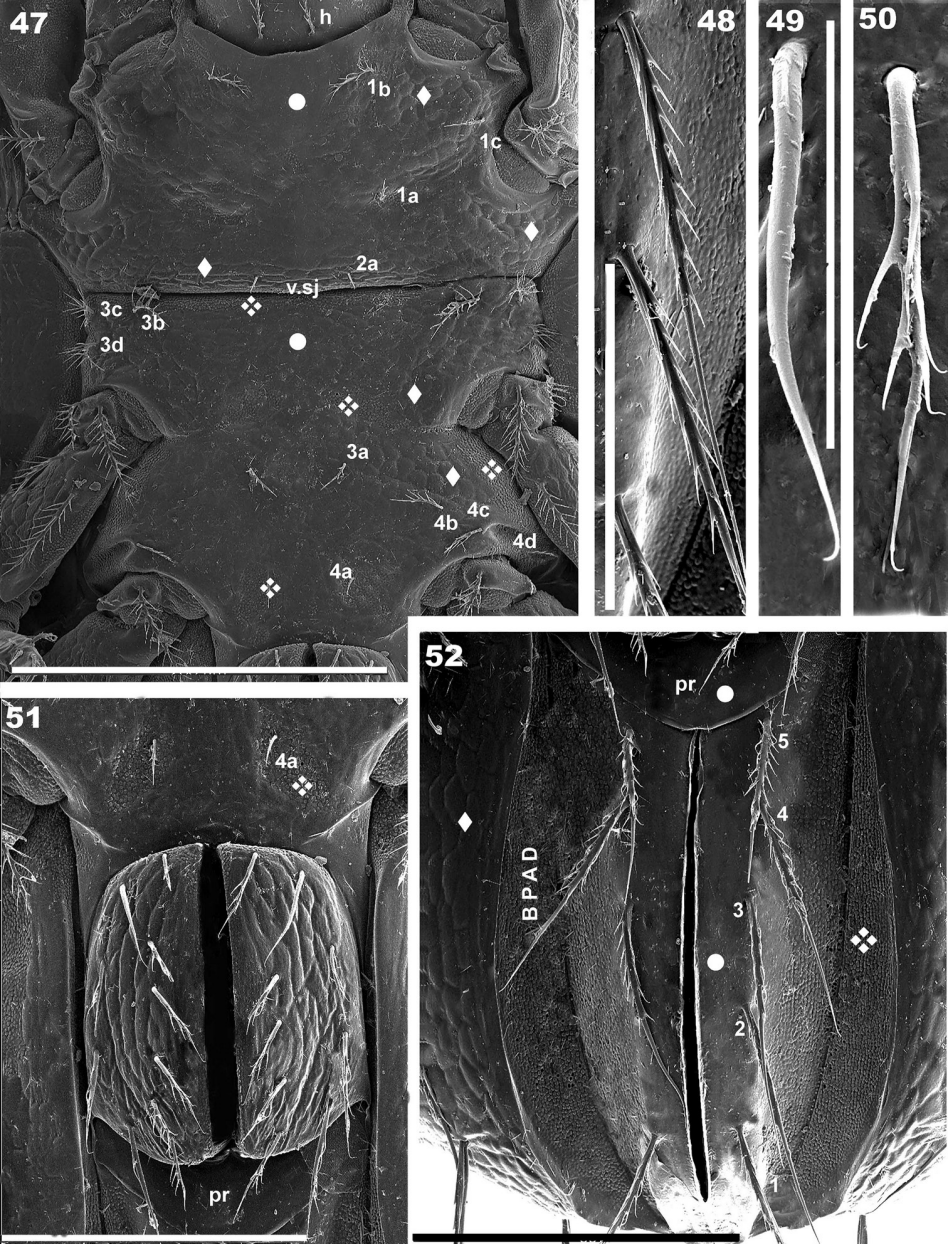
*Torpacarus eidikoterai* sp. n. Adult female with cerotegumental layer. SEM micrographs. **47** epimeral zone **48** adanal setae **49** genital setae, variation **50** genital setae **51** genital plate **52** anogenital zone. Scale bars: 200 μm (**47**); 50 μm (**48**); 7 μm (**49**); 7 μm (**50**); 100 μm (**51**); 100 μm (**52**).

**Figures 53–54.**
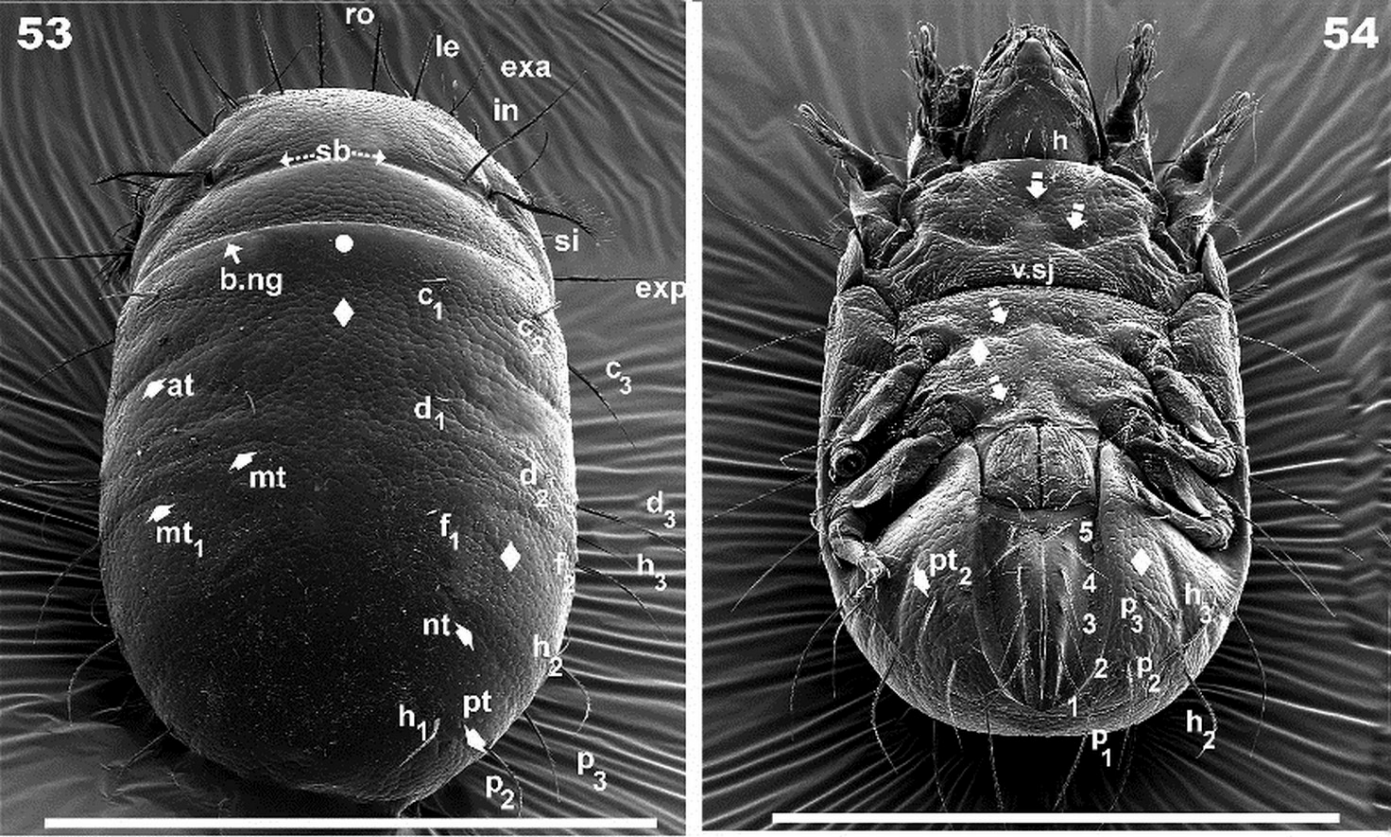
*Torpacarus eidikoterai* sp. n. Adult female with cerotegumental layer. SEM micrographs. **53** dorsal anteroposterior view **54** ventral anteroposterior view. Scale bars: 500 μm.

**Figures 55–58.**
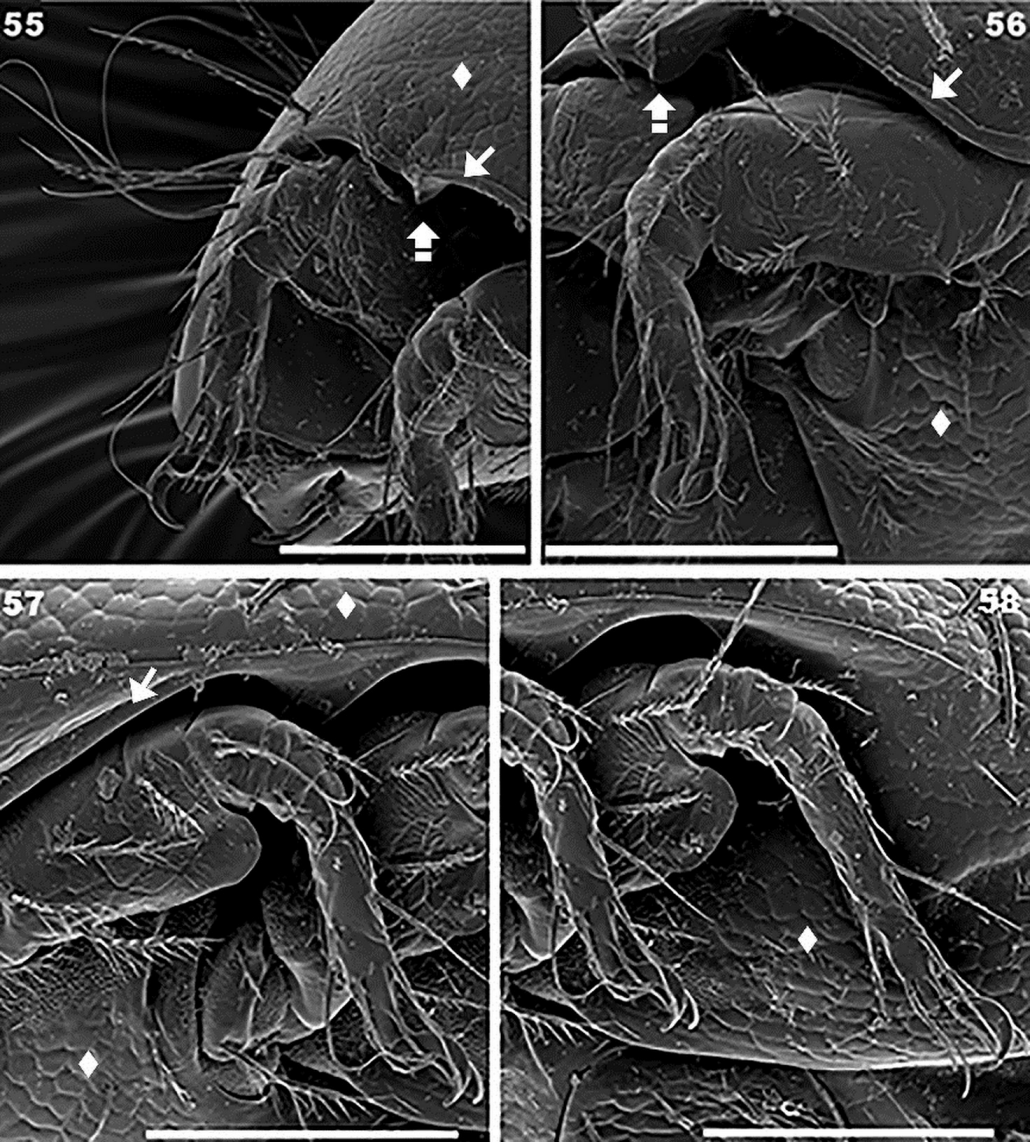
*Torpacarus eidikoterai* sp. n. Adult female with cerotegumental layer. SEM micrographs. **55** leg I, paraxial view **56** leg II, ventral paraxial view **57** leg III, paraxial view **58** leg IV, paraxial view. Scale bars: 100 μm (**55**); 100 μm (**56**); 100 μm (**57**); 100 μm (**58**).

*Integument.* Microsculpture varying according to body region: *areolate* (Figures 39, 53 indicated by diamond symbol): entire prodorsum (Figures 29, 35, 53, 55, indicated by diamond) besides medial anterior zone of CSO (Figures 39, 40, indicated by diamond see below); entire notogaster (Figures 29, 35, 37, 38, 53, 54 indicated by diamond) lateral to BPAD near setal insertion p_2_, p_3_ extending to IV acetabulum (Figures 37, 42, 52, 54, indicated by diamond); epimeral zone (Figures 42, 47, 54, 56, 57, 58, indicated by diamond). *Smooth* on anterior prodorsal zone of CSO (Figures 39, 40, indicated by large dot); anterior notogastral zone (Figures 29, 35, 38 indicated by large dot); anterior epimeral zone (Figure 47, indicated by large dot), central epimeral zone behind v.sj furrow (Figure 47 indicated by large dot); subcapitulum (Figures 45, 46 indicated by large dot); preanal zone and internal zone adanal plate (Figure 52 indicated by large dot); *colliculate* epimeral zone at level of acetabulum IV (Figure 47, indicated by diamond made up of 4 smaller diamonds); around 4a epimeral setal insertion (Figure 51 indicated by diamond made up of 4 smaller diamonds); lateral adanal zone and BPAD (Figure 52 indicated by diamond made up of 4 smaller diamonds); *elevated ridges* on genital plate (Figure 51).

*Setation* (legs not included). Two types of setae: smooth and barbate: subcapitular setae a, adoral setae, or_1_, or_2_, or_3_ (Figures 45, 46). The second type consists of different sub-types: *large seta with large barbs*: prodorsal setae, notogastral setaec_3_, d_3_, e_2_, f_1_, f_2_, h_1_, h_2_, h_3_, p_1_, p_2_, p_3_; adanal setae (Figures 30, 31, 34, 35, 36, 37, 38, 39, 40, 42, 48, 52). *Small setae with few barbs or nail-shaped*: notogastral setae c_1_, c_2_, d_1_, d_2_, e_1_ (Figures 32, 33). *Small setae with a few long barbs*: epimeral setae (Figure 44); genital setae (Figure 50), in some instances genital setae are observed lacking barbs, with feint dentition or smooth (Figure 49). *Medium length setae with barbs aligned on either side*: subcapitular setae h, m_1_, m_2_ (Figure 43), sometimes limited dentition between the two setal alignments.

*Prodorsum.* Polyhedral in dorsal view, between bng and le setal insertion levels (Figure 29, 36); anterior zone between le setal level and rostrum beak-shaped (Figures 29, 36).Lateral view triangular to polyhedral (Figures 35, 38). Polyhedral in frontal view between bng and le setal insertion level, elongate between le setal insertion level and rostrum (Figure 39), ro setae and CSO observed in elongate zone; typical areolate microsculpture also present; however in the medial anterior apical zone (forward ro setae) an elevated smooth longitudinal zone and CSO are observed (Figures 39, 40 indicated by large dot). Rostrum weakly bilobate with a small central structure (Figures 39, 40 indicated by solid upwards arrow); flat elevated margin extending dorsally, observed laterally up to smooth elevated longitudinal zone, terminating near rostrum (Figures 39, 40 indicated by solid leftwards arrow) on either side of prodorsal area, externally to exa, exp, le, ro setae, derived from margin of lateral depression housing legs; ro setal length 57 (55–59) μm, erect, apically curving backwards (Figures 39, 40); le 92 (90–94) μm, erect, directing upwards (Figures 38, 39); exa 31 (32–34) μm, directing externally and upwards (Figures 38, 39); exp 90 (87–92) μm, externally directed (Figures 38, 39); in 89 (98–79) μm , erect, directing upwards, slightly backwards (Figures 38, 39).

Ovoid, ring-shaped bothridium (bo), slightly elevated from cuticular surface (Figures 30, 35, 38, 39, 41); lateral opening. Internal bothridial ring structure pronounced on cuticular surface (Figures 30, 41). Sensillus (Si) length: 67 (60–86), bipectinate (Figures 30, 35, 36) with 15–21 large pectines on one side and small on the other (Figures 30, 41), sometimes small pectines are difficult to observe on Si stem (Figures 30, 35, 36, 41). Post bothridial transverse band sb forming shallow groove, posterior to bo, setae in situated on groove margin (Figures 29, 35, 36, 38); conspicuous in dorsolateral (Figure 30), frontal (Figure 39) and dorsoposterior view (Figure 53).

*Notogaster.* Sixteen pairs of primary notogastral setae: c_1_, c_2_, c_3_, d_1_, d_2_, d_3_, e_1_, e_2_, f_1_, f_2_, h_1_, h_2_, h_3_, p_1_, p_2_, p_3_ clearly discernible (Figures 29, 35, 36, 38, 53). Small notogastral setae, usually barbate c_1_, c_2_, d_1_, d_2_, e_1_ length 37 (36.5–38) μm; sometimes seta e modified to nail-shaped: 18 (17–19) μm; large notogastral setae c_3_, d_3_, e_2_, f_1_, f_2_, h_1_, h_2_, h_3_, p_1_, p_2_, p_3_: 106 (105–108) μm. Transversal bands of the type described in *Meristacarus* not observed, however clearly visible depressions in dorsoposterior-anterior view (Figure 53), with similar positioning of band indicated by Grandjean (1950) (see Remarks); depression mt behind d_2_,d_3_ setal insertion; oblique depression nt behind f_1_, h_2_setal insertion; pt depression behind h_1_ setal insertion level (Figure 53).

Three other depressions: at situated behind c_2_, c_3_setal insertions; mt_1_ situated parallel to mt; pt_2_ only visible in ventro posterior-anterior (Figure 54) view, situated between h_2_, h_3_ setae (Figure 53). None of these depression crossing medial notogastral plane.

Five pairs of lyrifissures present: ia, ip, im, ip, ih and ips; im behind d_2_, d_3_ (Figure 35); ip behind f_2_ (Figure 37); ia at level of c_3_setal insertion (Figure 37); ih anterior to h_2_ setal insertion; ips situated on the adanal fold band (BPDA) (Figure 35).

Posterior anterior view. Dorsoposterior-anterior view (Figure 53). Bulged, distended body shape. All transversal depressions easily observed: bd, b.ng, at, mt, mt_1_, nt, pt.

Ventral posterior-anterior view (Figure 54). Epimeral depressions: three paired and one unpaired ; depression *pt_2_*clearly visible (indicated by upwards white bar arrow).

*Lateral region.* Only transversal prodorsal band *sb* and notogastral depressions mt_1_, pt, pt_2_ (Figure 38) discernible. Flat smooth elevated margin, derived from lateral depression housing legs (Figures 38, 39, 40 indicated by solid leftwards arrow), extending to leg I-III (Figures 38, 55, 56, 57), clearly visible. Large spur present between legs I and II (Figures 38, 55, 56 indicated upwards white bar arrow). Anterior notogastral zone with conspicuous tectum and clearly defined unsclerotized lateral longitudinal line (Figures 35, 38) extending to h_3_ setal insertion level. Where unsclerotized line absent, notaspis and pleuraspis not delimited (Figures 35, 38) on posterior notogastral zone.

*Ventral region*. Subcapitulum polyhedral, posterior zone ovoid; spur visible behind subcapitular setae m_2_ insertion level, in marginal position (Figure 45) in an area with *colliculate* microsculpture (Figure 45 indicated by large diamond made up of 4 diamonds). Four pairs of subcapitular setae a, m_1_, m_2_, h. Length a: 44 (42–46) μm; h: 39 (38–40) μm; m_1_: 33 (31–35) μm; m_2_: 42 (38–46) μm.

Adoral setae (Figures 45, 46): or_1_ large, teardrop-shaped; or_2_ elongate, wide, terminating in acute end; or_3_ long and thin, sharply tipped. Length: or_1_: 30 (29–2) μm; or_2_: 42 (43–45) μm; or_3_: 20 (19–22) μm.

Coxisternal region divided into two parts by ventrosejugal groove (Figures 42, 47). Apodemes clearly visible; most of epimeral zone integument smooth (Figures 42, 47 indicated by large dot); areolate microsculpture in marginal zones posterior to acetabulum I-IV, and anterior to v.sj groove (Figure 42, 47 indicated by diamond). Zone posterior to acetabulum III, v.sj groove and some epimeral zones *colliculate* (Figure 47 indicated by diamond made up of 4 diamonds). Epimeral setal formulae 3-1-4-4) (Figure 47). Length of setae: 26 (23–29) μm; barbs of epimeral setae: 6 (4.3–7.5) μm. Genital plate undivided, rounded; ten pairs of setae, some instances asymmetric with only nine on one side; microsculpture of *elevated ridges* (Figure 51). Setal length: 32(31–34) μm. Preanal plate smooth, more or less triangular, rounded in central zone (Figure 52 indicated by large dot). Central zone of adanal plate *smooth* (Figure 52 indicated by large dot); zone near BPAD and BPAD *colliculate*; adanal setae length: 81(79–91) μm ; BPAD band clearly visible; lyrifissure ips present near band margin (Figures 37, 42, 52).

*Legs* (Figures 55–58). Two types of femora distinguished. Femora legs I and II displaying underdeveloped ventral blade (Figures 55, 56); femora legs III and IV with large ventral blade (Figures 57, 58). Setal formulae I (0-4-3-4-15-1) (2-1-2); II (0-5-3-4-13-1) (1-1-1); III (2-4-2-2-11-1) (1-1-0); IV (2-3-2-2-10-1(1-0-0).

###### Remarks.

Porose areas were not observed. Shallow depressions indicated by Grandjean (1950) as mt, nt and pt are present, three further depressions were observed: at situated in front of mt; mt_1_ behind mt; and pt_2_ behind pt; pt_2_ is a deep depression, clearly visible with optical microscopy and SEM. In *Torpacarus omittens* Grandjean (1950), mt and nt cross the medial notogastral plane; however, in *T. eidikoterai* sp. n. none of the observed depressions observed cross the medial notogastral plane.

## Discussion

*Meristacarus perikopesis* sp. n. is close to *Meristacarus porcula* Grandjean, 1934. Some characters were compared using optical microscopy, while detailed observations using SEM aided in providing certainty in comparison of structures. A series of shared characters were observed such as: body shape; numerous porous areas on prodorsum, notogaster and epimeres (“pusticulate surfaces” see Remarks); presence of ten transversal bands. Some observed differences in comparison to other congeners: shape of porose areas; differences in position of prodorsal *in* setae, which in *Meristacarus perikopesis* sp. n. are situated near bo and on the sb band; large smooth zone in front of S_1_ and b.ng; barbate prodorsal and notogastral setae; transversal bands S_4_, S_5_, S_7_, S_10_ not crossing medial notogastral plane; differences in shape of adoral setae or_1_.

Adequately comparing *Torpacarus eidikoterai* sp. n. is impeded by the lack of detailed figures, besides dorsal and ventral views, in descriptions of other congeners. The shape of the prodorsum in *Torpacarus eidikoterai* sp. n. differs from all fifteen other species with the exception of *T. gramineus* McDaniel, Norton & Bolen, 1979 (figure 4 page 627) where the rostrum appears bilobate, though the authors did not note this in text.

Other important aspects for comparison include the sizes of some central notogastral setae, and the transversal lines (or depressions). Setae c_1_, c_2_, d_1_, d_2_, e_1_ are small in *T. eidikoterai* sp. n and at, mt, mt_1_, nt, pt, pt_2_ transversal depressions are present. This can be compared to: *T. foveolatus* Wallwork, 1962, with small c_1_, d_1_, d_2_, e_1_ setae and medium sized c_2_, f_1_, h_1_, where all setae are mostly smooth (occasionally with barbs), and transversal lines are not present. *T. magnus* Wallwork, 1962 presents small setae c_1_, d_1_, d_2_, e_1_ with other setae of normal size, and only transversal line mt present; *T. cinctus* Wallwork, 1962, small setae c_1_, d_1_, d_2_, e_1_, transversal lines mt and nt present; *T. omittens* Grandjean, 1950, setae c_1_, d_1_, e_1_small;transversal lines mt, nt, pt present. Finally *T. remotus* Schatz, 1994 with small c_1_, c_2_, d_1_, d_2_, e_1_ and medium sized f_1_, h_1_ setae; transversal bands mt, nt, pt present. The ensemble of characters clearly distinguishes *Torpacarus eidikoterai* sp. n. from other congeners.

## Supplementary Material

XML Treatment for Meristacarus perikopesis

XML Treatment for Torpacarus eidikoterai
